# A Table-Shaped Tactile Sensor for Detecting Triaxial Force on the Basis of Strain Distribution

**DOI:** 10.3390/s131216347

**Published:** 2013-11-28

**Authors:** Jeong Il Lee, Min-Gyu Kim, Mitsuhiro Shikida, Kazuo Sato

**Affiliations:** 1 Department of Micro-Nano Systems Engineering, Nagoya University, Nagoya 464-8603, Japan; E-Mail: shikida@mech.nagoya-u.ac.jp; 2 Department of Industrial Design, Eindhoven University of Technology, AZ Eindhoven 5612, The Netherlands; E-Mail: m.kim@tue.nl; 3 Department of Mechanical Engineering, Aichi Institute of Technology, Aichi 470-0392, Japan; E-Mail: sato@aitech.ac.jp

**Keywords:** tactile sensor, table-shaped structure, decoupling of triaxial force

## Abstract

A slim and flexible tactile sensor applicable to the interaction of human and intelligent robots is presented. In particular, a simple sensing principle for decoupling of three-dimensional force is proposed. Sensitivity of the proposed tactile sensor is tested experimentally. To improve the sensitivity of the sensor, a table-shaped sensing element was designed. Table-shaped structure can convert an external acting force into concentrated internal stress. A “triaxial force decoupling algorithm” was developed by combining two-dimensional mapping data calculated by finite element analysis. The sensor was calibrated under normal and tangential forces. The external loads applied to the sensor could be decoupled independently as a function of the strain-gauge responses.

## 1. Introduction

In recent years, studies on human–robot interaction have needed to utilize tactile sensors to physically interact with people and their environment in contexts such as rehabilitation, home/hospital care, education, and entertainment. In regard to human communication, physical touch is essential for an infant or child's social, cognitive, and physical development [1]. Touch also plays an important role in adulthood, when a person is soothing, playing, and maintaining proximity between a child and caretaker [2]. Similarly, physical interaction with robots (such as hugging and hand shaking) builds closer relationships between a human and a robot from the perspective of spatial distance [3]. The ROBOSKIN project shows another application domain of tactile interaction, namely, that between robots and autistic children to improve social-interaction capabilities of the children [4]. Furthermore, physical therapy for stroke rehabilitation by robotic manipulators is a promising application of tactile sensors [5].

In work on the human–robot collaboration, researchers have been developing tactile sensors [6,7]. Manipulating objects by a robotic hand requires flexible tactile sensors mounted on the curved surface of the robot's fingertip to detect the forces acting on it. To dexterously manipulate and grasp an object while maintaining physical contact between the object and the robot's finger tips, it must be possible to measure normal and tangential forces acting on the object. To enable that measurement, two studies applied displacement control by robot-vision systems [8,9] and another used a force-toque sensor on the fingertips of the robotic hand [10]. In consideration of the role of tactile sensors in the domain of human–robot interaction, it is necessary to ensure that the sensors are small and flexible enough to fit onto various surfaces of machinery components as well as have sufficient accuracy to allow precise manipulation of the robotic hand.

Researchers have been developing several types of tactile sensor based on “microelectromechanical systems” (MEMS) composed of different materials (e.g., silicon and polymers) because MEMS can be applied to tiny integrated sensing units. In the early stage of developing tactile sensors, Kane *et al.* and Mei *et al.* used micromachining to fabricate diaphragm-style tactile sensors composed of triaxial force sensors [11,12]. Although these silicon micro-machined tactile sensors have high sensitivity, they are not flexible or durable. Silicon is mechanically brittle, so it cannot sustain large deformations and sudden shocks. Additionally, a rigid silicon substrate makes it difficult to cover contoured surfaces [13,14]. The characteristics of silicon-based tactile sensors makes it difficult to apply them in wide variety of domains and, at the same time, increases development cost. Meanwhile, other approaches are using change of capacitance or conductive polymer films [15–17]. Even if these types of tactile sensors are beneficial in terms of low development cost and higher flexibility, they still have two drawbacks: low sensitivity and poor spatial resolution. To create a tactile sensor with both small size and high flexibility, polymer micromachining (which makes it possible to integrate MEMS devices on a flexible polymer substrate) has been introduced. Engel *et al.* have proposed a “flexible tactile-sensor skin” that has an expandable sensor array [14]. However, this sensor (skin) is can only measure a normal force component. To overcome these drawbacks of the silicon-based and polymer-based approaches, “hybrid tactile sensors”—combining silicon and polymers—have recently been developed [18–20]. Various other types of tactile sensor, including optical ones, have been developed [6,21]. Although flexible polymer-based tactile sensors are being increasingly reported, it is still a great challenge to implement three-axial force detection with a small tactile sensor. In a previous study, the authors demonstrated a prototype flexible tactile sensor with a three-dimensional table-shaped structure [22]. However, we did not present a solution to the problem of simultaneously applying a three-dimensional resultant force; that is, our previous work presented measurement results under the restriction of independently separated normal and tangential forces [22]. Generally, if the resultant force is applied at a point on the contact surface, it is possible to apply torque to the contact surface. In the present study, this difficulty is irrelevant since the target of the sensor is assumed to be manipulated by the robot's finger. In other words, torque does not occur because loading applied to the contact surface is a uniformly distributed load (*i.e.*, not a point). However, we plan to improve the algorithm for decoupling the applied three-dimensional force by assuming that torque is exerted by the applied force. In the present study, a new approach for decoupling the applied three-dimensional force by using normalized force components is therefore proposed. In particular, the mechanism of force detection was identified, and a decoupling algorithm for a tactile sensor was devised and applied to the dexterous manipulation by a robotic hand.

## 2. Triaxial Force Decoupling

### 2.1. Sensor Body and Circuit Design

**Figure 1.**
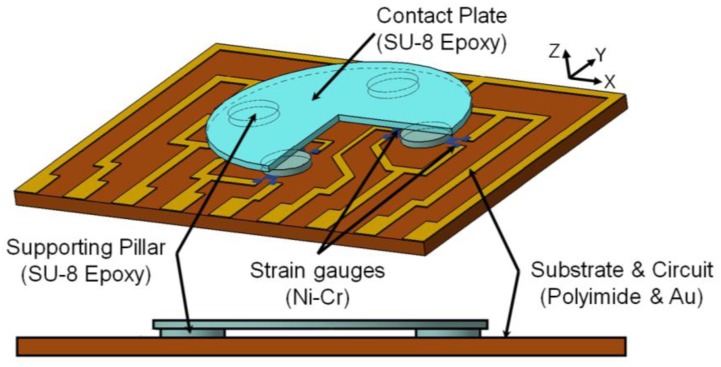
Schematic diagram of the designed sensor and cross-sectional view of the sensing unit.

In this study, a resistance-type tactile sensor is used. A strain gauge can convert an external force to change of resistance as an internal strain. To amplify a contact stress, a tactile-sensing pad has a three-dimensional, small and thin structure with a table-shaped top-head. A schematic diagram and cross-sectional view of the table-shaped sensing pad is shown in Figure 1. A polymer material (SU-8 epoxy) was used as the three-dimensional structure of the contact plate and force-transfer pillars.

To maximize the sensitivity of the sensor, the optimal locations of the strain gauges were determined by the strain distribution obtained by finite element analysis. The strain distribution was then used to determine the shape of the strain gauge and its size. Configuration of strain gauges is carefully investigated to set the area of highest strain. The conceptual design of the sensor was determined by a commercial finite element analysis (FEA) program, *i.e.*, ABAQUS Ver. 6.10.

Since the external force applied to the sensing plate is transmitted to the substrate through the force-transfer columns, most strain changes on the substrate appear on the bottom of the strained columns. From the FEA analysis results, it is clear that the strain-sensing elements in the tactile sensor must be placed at the periphery of the columns. The designed tactile sensor consists of a 60-μm-thick, 1,870-μm-diameter upper plate as a sensing element and four 60-μm-high, 440-μm-diameter force-transfer columns on a 125-μm-thick, 4.18 × 2.91-mm rectangular membrane (see Figure 1). The membrane material is a polyimide film (with Young's modulus of 2.5 GPa and Poisson's ratio of 0.34). For the column and upper-plate materials, SU-8, with Young's modulus of 4.4 GPa and Poisson's ratio of 0.22, is used [23].

**Figure 2.**
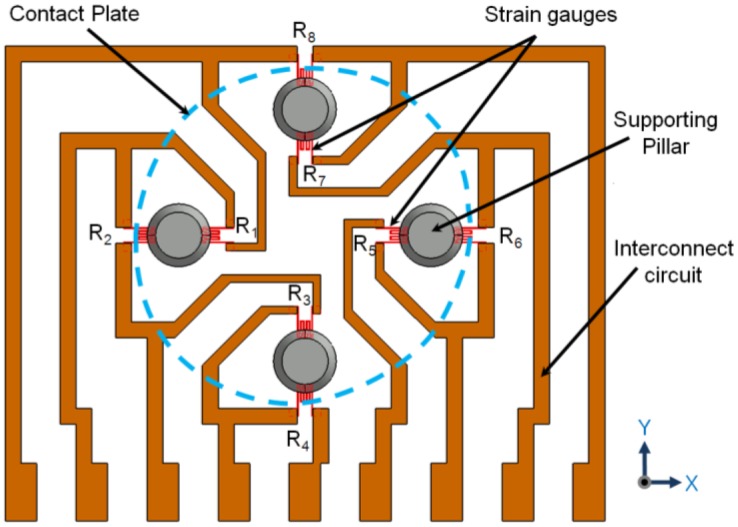
Schematic representation of the configuration of the resistor and interconnects. Eight strain gauges are candidates for quarter-bridge or moment-compensation circuit. Four strain gauges (R_1_, R_3_, R_5_, and R_7_) were used to measure applied normal and 2D tangential forces.

As shown in Figure 2, four strain gauges (R_2_, R_1_, R_5_, and R_6_) along the *x*-axis and four more strain gauges (R_4_, R_3_, R_7_, and R_8_) along the *y*-axis are arranged as pairs under the four force-transfer columns. The resistance of the strain gauges is changed symmetrically as they are physically deformed according to the applied direction of external force. Put simply, to measure the direction and magnitude of the applied external force, just four strain gauges (R_1_, R_3_, R_5_, and R_7_) inside of the contact plate are needed. The direction and magnitude of applied force could therefore be measured by combination of either increasing or decreasing the output resistance.

### 2.2. Superposition Principle of Vector Force

Force applied to a monolithic sensor is a vector combination of normal and tangential forces. The mixed components of normal and tangential forces must be decoupled. However, it is a problem that the decoupling cannot be done directly. To solve this problem, output resistance can be related to applied loading. A distribution map of the strain gauges (which depends on the configuration of the gauges) can then be drawn. It is therefore possible to approximately calculate the direction and magnitude of the applied loading through the reconstruction of the contributing directional forces (which are combined by using calibration data).

**Figure 3.**
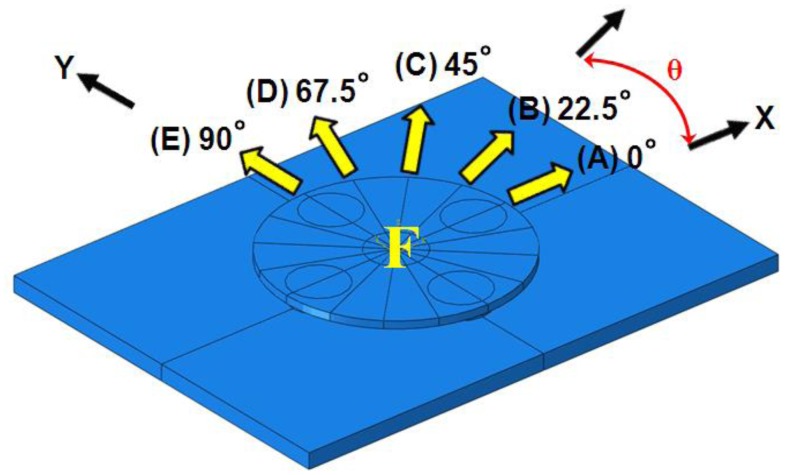
Schematic representation of the design of loading simulations for each directional tangential loading.

**Figure 4.**
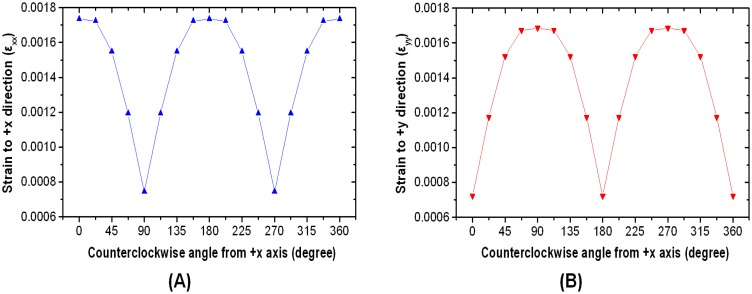
(**A**) Maximum strain change under 1-N directional tangential loading against principal strain variation in the +*x* direction (ε_xx_) and (**B**) principal strain variation in the +*y* direction (ε_yy_).

**Figure 5.**
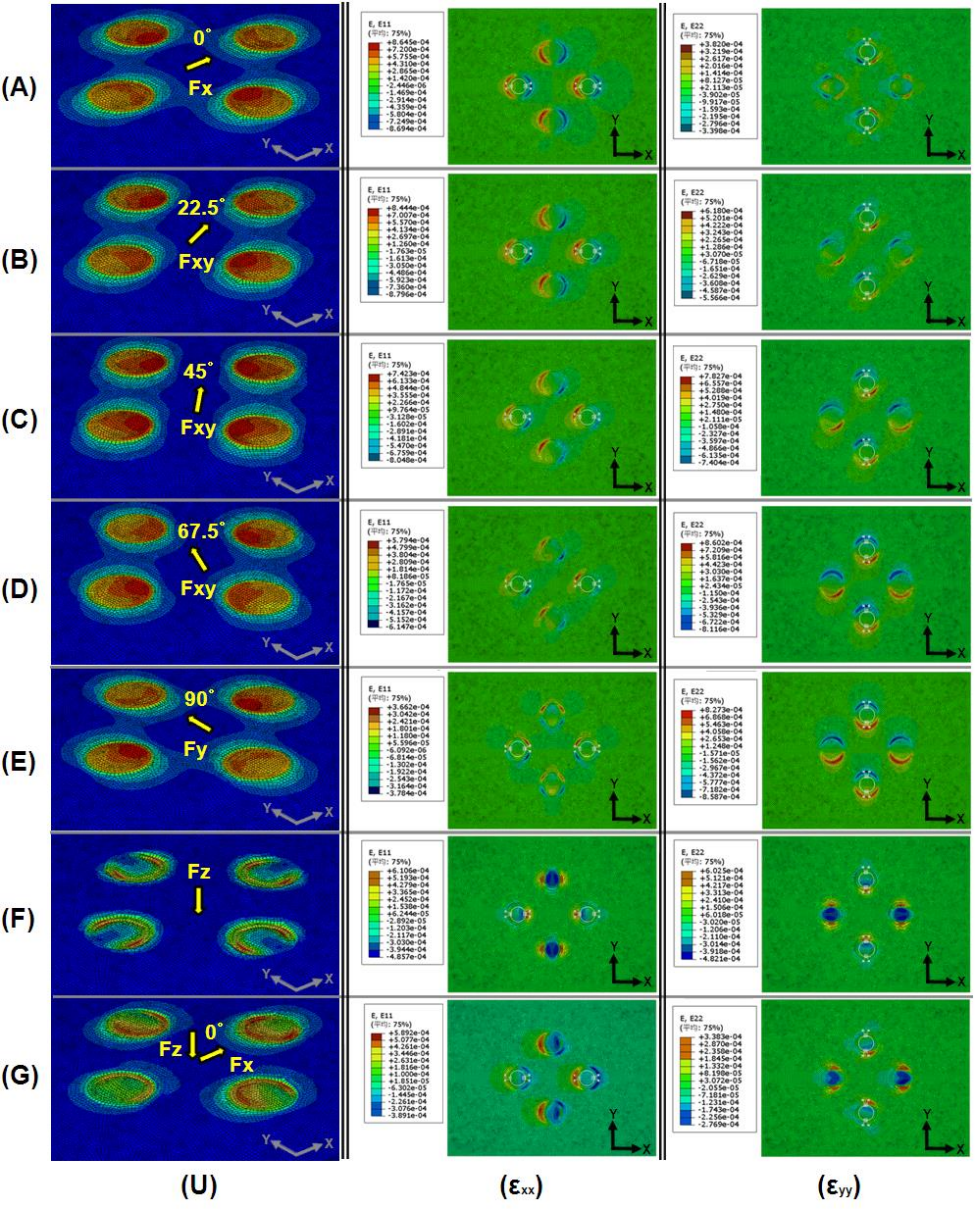
FEM results of loading simulations. Displacement contour (U) and (ε_xx_), (ε_yy_) strain distribution due to application of 1-N directional tangential loading at (**A**) 0°; (**B**) 22.5°; (**C**) 45°; (**D**) 67.5°; and (**E**) 90° against the +*x*-axis, respectively; (**F**) Displacement contour (U) and (ε_xx_), (ε_yy_) strain distributions due to application of 1-N normal loading; (**G**) Resultant force of normal and tangential loading at 0° against the +*x*-axis. Notice the configuration of the strain gauges lies along the principal strain directions ((ε*_xx_*), (ε*_yy_*)).

A schematic representation of the design of loading simulations for each directional tangential loading is shown in Figure 3. Maximum strain change under 1-N directional tangential loading is plotted against principal strain variation in the +*x* direction (ε_xx_) in Figure 4A, and principal strain variation in the +*y* direction (ε_yy_) is plotted in Figure 4B. These data are measured every 22.5° from 0° to 360° parallel to the +*x*-axis. These asymmetric sinusoidal results caused by the configuration of the support pillar could be expandable in two-dimensional mapping shown as Figure 5. We are able to produce a two-dimensional strain distribution map to directional loading divided into five cases.

The results of FEA loading simulations, namely, displacement contour (U) and strain distribution due to application of 1-N directional tangential loading [(ε_xx_) and (ε_yy_)] at 0°, 22.5°, 45°, 67.5°, and 90° against the +*x*-axis, are plotted in Figure 5A–E, respectively. A displacement contour (U) and strain distribution due to application of 1-N normal loading [(ε_xx_) and (ε_yy_)] are plotted in Figure 5F. A displacement contour (U) and strain distribution due to application of normal and tangential loading ((ε_xx_) and (ε_yy_)) at 0° against the +*x*-axis are plotted in Figure 5G. To find the origin of the applied force, we are able to use two-dimensional mapping data obtained from the directional force contribution of the strain distribution. Direction and magnitude of the applied loading can therefore be back-calculated by reconstructing an image of the total-axial-force components. This superposition principle is theoretically right (at least in the elastic region).

### 2.3. Decoupling Method

Increases and decreases in change of resistance according to direction of applied external force are listed in Table 1. In the table, “(+)” and “(−)” signs indicate increase and decrease in resistance against the initial value, respectively. For example, when an external force is applied in the +*x*-axis direction, strain gauges R_1_ and R_5_ adopt compressive and tensile states, respectively. The resistances therefore decrease (−, R_1_) and increase (+, R_5_), respectively. Meanwhile, when an external force is applied in the −*x*-axis direction, strain gauges R_1_ and R_5_ adopt tensile and compressive states, respectively. These resistance changes are opposite to those in the case that the external force is applied in the +*x*-axis direction. Note here that R_3_ and R_7_ lie along the *y*-axis and the change of resistance is negligibly small (see strain distribution (ε_yy_) in Figure 5A). When an external force is applied in either the ±*x* or ±*y*-axis direction, the other axial change of resistance should be designed to be as small as possible. When an external force is applied in the +*y*-axis direction, the resistance changes of R_3_ and R_7_ are decreased and increased, respectively. Here, R_1_ and R_5_ (lying along the *x*-axis) are also not physically changed (see strain distribution (ε_xx_) in Figure 5E). On the other hand, when an external force is applied in the normal direction (*i.e.*, the −*z*-axis direction) relative to the contact plate surface, the resistances of all the strain gauges (R_1_, R_3_, R_5_, and R_7_) increase (see Figure 5F).

The strain distributions due to the configuration of the strain gauges give the decoupling solution derived from the directional force components in the following equations. Generally, this decoupling solution has been widely applied to resistance-type tactile sensors [24–27]; for −F_z_ loading; from strain gauge signals of R_1_, R_3_, R_5_ and R_7_:

(1) $$\Delta R(F_{-z})=\Delta R\left(R_{1}\right)+\Delta R\left(R_{3}\right)+\Delta R\left(R_{5}\right)+\Delta R\left(R_{7}\right)$$

for +F_x_ loading; from strain gauge signals of R_1_ and R_5_:

(2) $$\Delta R\left(F_{+x}\right)=\Delta R\left(R_{1}\right)-\Delta R\left(R_{5}\right)$$

for +F_y_ loading; in a similar way:

(3) $$\Delta R\left(F_{+y}\right)=\Delta R\left(R_{3}\right)-\Delta R\left(R_{7}\right)$$

Here, ΔR means the change of resistance of the strain gauge. The positive sign represents a tensile state, and the negative sign represents a compressive state. From Equations (1) to (3), the magnitude of loadings can be calculated. Applied loadings along the *x*-, *y*-, and *z-*axes can be estimated through calculation of the resistance change from the output signals of the four strain gauges arranged inside contact plate. In Figure 2, the four extra strain gauges (R_2_, R_4_, R_6_, and R_8_) arranged outside of the contact plate (which are candidates for a quarter-bridge or moment-compensation circuit) are also shown. However, these extra strain gauges are not considered in this study.

### 2.4. Application of Decoupling Method to Loading Simulations

**Figure 6.**
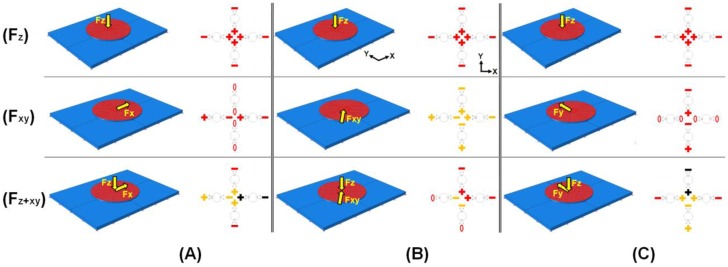
(**A**) Strain distribution under applied normal load, +*x*-direction (0°) tangential load, and superposition of normal and tangential load; (**B**) Strain distribution under applied normal load and diagonal-direction (45°) tangential load and superposition of normal and tangential load; (**C**) Strain distribution under applied normal load and +*y*-direction (90°) tangential load and superposition of normal and tangential load.

The combined results of Figures 4 to 5 and Table 1 are shown in Figure 6A–C. The FEA simulation result for superposition of normal force and tangential force at 0° parallel to the +*x*-axis is shown in Figure 6A. When a normal load is uniformly applied to the top surface of the contact plate, the simulation result shows a symmetrical feature under the support column. Four strain gauges inside the contact plate undergo tensile state (see Figure 6F_z_). However, the state of the four strain gauges arranged outside the contact plate becomes compressive. Meanwhile, in the case that a tangential load is uniformly applied to the contact plate, the simulation result shows two different results. The state of the strain gauges arranged in the forward direction of the columns parallel to the loading direction becomes compressive, while the state of the strain gauges arranged in the backward direction of the columns parallel to the loading direction becomes tensile. Notice that the four strain gauges at a right angle to the loading direction do not change state (see Figure 6F_xy_). Therefore, superposition of normal force and tangential force at 0° parallel to the +*x*-axis shows the sum of the strain change at each strain gauge. However, tangential variation is more dominant compared to normal variation in the case of the resultant force, if the magnitudes of the applied normal and tangential forces are the same.

Accordingly, when the positive sign of a tangential force meets the negative sign of a normal force, it does not give zero (see Figure 6F_z+xy_). In the figure, red indicates the strain change values under applied loading of 1 N. Black indicates duplicated red. Yellow indicates counterbalanced value when (+) and (−) red meet each other. Similarly, results for the superposition of normal force and tangential force at 45° and 90° parallel to the +*x*-axis are shown in Figure 6B,Crespectively. The signal combinations of all possible arrangements of the strain gauges and the predicted direction are listed in Table 2. The directions of applied tangential force can be predicted by comparing the configuration of settled standard feature using Figure 6 due to Table 1 and Figures 4 and 5. Prediction resolution is shown as three levels of color (*i.e.*, black, red, and yellow). Note that the prediction resolution can be increased by increasing the number of color levels.

## 3. Sensor Fabrication

**Figure 7.**
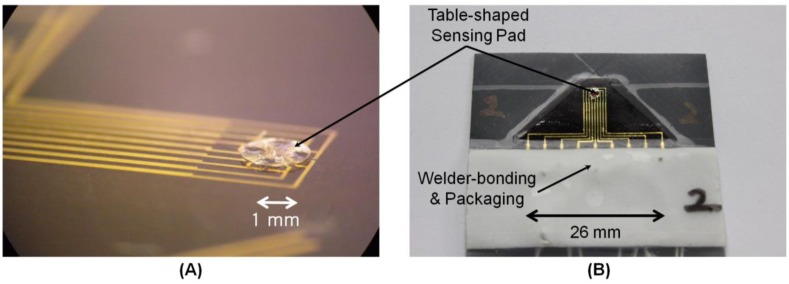
(**A**) Fabricated sensor body and table-shaped sensing pad and (**B**) Packaged tactile sensor after welder bonding.

A tactile sensor was fabricated (inside a clean room) by the following process: first, nickel-chrome-alloy (Ni-Cr; 80:20) strain gauges were made by sputtering and lift-off techniques. In detail, a 400-Å-thick Ni-Cr layer was patterned on a 100-Å-thick chromium adhesion layer on a 125-μm-thick polyimide substrate. Then, 2,000-Å-thick gold interconnects were deposited on a 200-Å-thick chromium adhesion layer by sputtering and lift-off on the top surface of the polyimide film. A sacrificial layer was used to make a table-shaped three-dimensional structure as a sensing element. As a contact plate and support column respectively, SU-8 epoxy was deposited by using a photo-definable lithography process on the surface of the film. After all micromachining process was completed, the fabricated sensor (with a total thickness of 250 μm) has a radius of curvature of less than 8 mm. This curvature coincides with the expected flexibility of this study. The fabricated sensor body and table-shaped pad are shown in Figure 7A. Finally, the tactile sensor was connected to an electric cord and packaged (see Figure 7B).

## 4. Force Detection

The fabricated tactile sensor was evaluated by using a three-component load cell to apply normal and tangential forces between zero and 1 N, respectively. For the signal processing, a PCI-type 12-bit/16-channel data-acquisition board (PCI-3174, Interface. Co., Hiroshima, Japan) and I/O interface (TRM-7101, Interface. Co) were connected to the sensor with a power supply (IPS-3610D, ISO-tech. Co., Corby, UK), and a constant force was applied to the sensor. The voltage change of each strain gauge was converted to a resistance through the data-acquisition board, and the load was calculated from that resistance. While force was applied to the sensor along the z- and x-axes, the change in force on the four strain gauges, R_1_, R_3_, R_5_ and R_7_, was measured. The measured increases and decreases in resistances were found to be equal to the theoretically expected values listed in Table 1.

**Figure 8.**
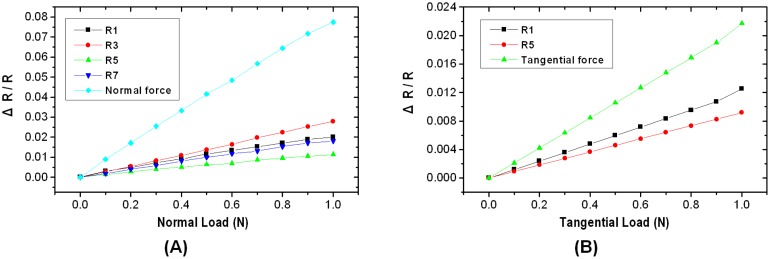
Normalized evaluation results change in output resistance under (**A**) normal loading F_z_ and (**B**) tangential loading F_x_.

When F_z_ loading increasing from 0 to 1 N was applied to the sensor, the output signal due to the resistance change with respect to the F_z_ loading (which is normalized by resistors R_1_, R_3_, R_5_, and R_7_) is shown in Figure 8A. Ideally, strain gauges made by the same process will have equal resistances. Although the experimental setup is perfect, fabrication errors due to the uncontrollable factors are inevitable. If the fabrication process is improved in the future, we believe that it will be possible to provide a simpler algorithm. The resolution of resistance with respect to force is measured at 77,465 ppm/N in terms of ΔR/R. In the case of applied F_x_ loading (which is normalized by resistors R_1_ and R_5_), the resolution of resistance with respect to force is measured at 21,693 ppm/N, see Figure 8B. To verify the proposed decoupling method, we plan to develop an experimental setup for applying normal and tangential loadings simultaneously.

## 5. Concluding Remarks

A new type of slim, flexible tactile sensor and an analytical approach for a decoupling method for obtaining directional three-dimensional force components were proposed. A table-shaped epoxy sensing plate with four legs was built on the top of a flexible polymer substrate to enhance sensor sensitivity. The applied forces could be calculated by combining the signal responses from the strain gauges. To calculate the applied loading, two-dimensional mapping results derived from finite element analysis was developed. The tactile sensor was calibrated to normal and tangential loadings from 0 to 1 N by the evaluation apparatus. The proposed sensing and decoupling algorithm are able to measure the triaxial forces only using four strain gauges. In the future, the sensing and decoupling algorithm will be improved to measure not only force, but also torque, by using all eight strain gauges and to solve a moment interference problem when a sensing pad meets the inclined force. It is hoped that the improved sensor will find many applications in the fields of not only intelligent robotic hand manipulation, but also physical human–robot interaction and human–robot collaborative tasks such as dependable force feedback control.

## Figures and Tables

**Table 1.** Increase and decrease of resistance according to direction applied external force.

		**Sign of the Strain Gauges by Applying Direction**				
		**+*x***	**−*x***	**+*y***	**−*y***	**−*z***
Strain gauges on the axis along the *x*-direction	**R_2_**	(+)	(−)	0	0	(−)
	**R_1_**	(−)	(+)	0	0	(+)
	**R_5_**	(+)	(−)	0	0	(+)
	**R_6_**	(−)	(+)	0	0	(−)

Strain gauges on the axis along the *y-*direction	**R_8_**	0	0	(−)	(+)	(−)
	**R_7_**	0	0	(+)	(−)	(+)
	**R_3_**	0	0	(−)	(+)	(+)
	**R_4_**	0	0	(+)	(−)	(−)

**Table 2.** The signal combination of all possible arrangements of the strain gauges and the predicted direction.

	**Signals and Decisions**				

	**R_1_**	**R_3_**	**R_5_**	**R_7_**	**Decision**
**1**	**+**	**+**	**+**	**+**	**F_−_***_z_*
**2**	**−**	**0**	**+**	**0**	**F_+_***_x_*
**3**	**+**	**0**	**−**	**0**	**F_−_***_x_*
**4**	**0**	**−**	**0**	**+**	**F_+_***_y_*
**5**	**0**	**+**	**0**	**−**	**F_−_***_y_*
**6**	**−**	**−**	**+**	**+**	**F_+_***_x_***_+_***_y_*
**7**	**+**	**−**	**−**	**+**	**F_−_***_x_***_+_***_y_*
**8**	**+**	**+**	**−**	**−**	**F_−_***_x_***_−_***_y_*
**9**	**−**	**+**	**+**	**−**	**F_+_***_x_***_−_***_y_*
**10**	**−**	**+**	**+**	**+**	**F_−_***_z_***+ F_+_***_x_*
**11**	**+**	**+**	**−**	**+**	**F_−_***_z_***+ F_−_***_x_*
**12**	**+**	**−**	**+**	**+**	**F_−_***_z_***+ F_+_***_y_*
**13**	**+**	**+**	**+**	**−**	**F_−_***_z_***+ F_−_***_y_*
**14**	**−**	**−**	**+**	**+**	**F_−_***_z_***+ F_+_***_x_***_+_***_y_*
**15**	**+**	**−**	**−**	**+**	**F_−_***_z_***+ F_−_***_x_***_+_***_y_*
**16**	**+**	**+**	**−**	**−**	**F_−_***_z_***+ F_−_***_x_***_−_***_y_*
**17**	**−**	**+**	**+**	**−**	**F_−_***_z_***+ F_+_***_x_***_−_***_y_*
